# Validation of a Spanish language version of the pain self-perception scale in patients with fibromyalgia

**DOI:** 10.1186/1471-2474-11-255

**Published:** 2010-11-04

**Authors:** J García-Campayo, B Rodero, Y López del Hoyo, JV Luciano, M Alda, M Gili

**Affiliations:** 1 Department of Psychiatry, Miguel Servet University Hospital, Instituto Aragonés de Ciencias de la Salud, University of Zaragoza, Zaragoza, Spain; 2 Clínica Rodero, Santander, Spain; 3 Department of Psychology, Instituto Aragonés de Ciencias de la Salud, University of Zaragoza, Zaragoza, Spain; 4 Parc Sanitari Sant Joan de Déu, Fundació Sant Joan de Déu, Sant Boi de Llobregat, Barcelona, Spain; 5 Institut Universitari d'Investigació en Ciències de la Salut (IUNICS), University of Balearic Islands, Palma, Spain

## Abstract

**Background:**

The Pain Self-Perception Scale (PSPS) is a 24-item questionnaire used to assess mental defeat in chronic pain patients. The aim of this study was to develop a Spanish language version of the PSPS (PSPS-Spanish), to assess the instrument's psychometric properties in a sample of patients with fibromyalgia and to confirm a possible overlapping between mental defeat and pain catastrophizing.

**Methods:**

The PSPS was translated into Spanish by three bilingual content and linguistic experts, and then back-translated into English to assess for equivalence. The final Spanish version was administered, along with the Hospital Anxiety Depression Scale (HADS), Pain Visual Analogue Scale (PVAS), Pain Catastrophizing Scale (PCS) and Fibromyalgia Impact Questionnaire (FIQ), to 250 Spanish patients with fibromyalgia.

**Results:**

PSPS-Spanish was found to have high internal consistency (Cronbach's α = 0.90 and the item-total *r *correlation coefficients ranged between 0.68 and 0.86). Principal components analysis revealed a one-factor structure which explained 61.4% of the variance. The test-retest correlation assessed with the intraclass correlation coefficient, over a 1-2 weeks interval, was 0.78. The total PSPS score was significantly correlated with all the questionnaires assessed (HADS, PVAS, PCS, and FIQ).

**Conclusions:**

The Spanish version of the PSPS appears to be a valid tool in assessing mental defeat in patients with fibromyalgia. In patients with fibromyalgia and Post-Traumatic Stress Disorder (PTSD), PSPS-Spanish correlates more intensely with FIQ than in patients without PTSD. Mental defeat seems to be a psychological construct different to pain catastrophizing.

## Background

Mental defeat (MD) has been defined as the perceived loss of autonomy in the face of uncontrollable traumatic events, resulting in the person giving up efforts to retain identity and self-will [1,2]. It has been implicated in the development and maintenance of Post-Traumatic Stress Disorder (PTSD) [1]. In the context of torture or assault, mental defeat predicts not only the development and severity of PTSD, but also the response to subsequent treatment [1-4]. This construct has also been strongly associated with the severity of depression, and the association remains highly significant even when hopelessness is controlled for [5].

As both PTSD and depression are frequently comorbid with chronic pain, Tang et al [6] studied the concept of mental defeat in chronic pain patients. They developed a specific questionnaire to measure it, the Pain Self-Perception Scale (PSPS), which showed adequate psychometric properties. This questionnaire has not been validated in other languages different from English, and it has not been used in fibromyalgia (FM), a chronic pain disorder".

One of the problems in the development of the construct "mental defeat" was its possible overlapping with another psychological construct that is considered relevant in chronic pain, pain catastrophizing (PC). PC is considered a continuous psychological variable, normally distributed even in healthy individuals without pain or depression [7]. It has been described as an exaggerated negative orientation toward noxious stimuli [8]. Tang et al. [6] theoretically distinguished mental defeat from catastrophizing, because the former is a type of catastrophizing focused not on the experience and meaning of pain *per se*, but rather on the effects of pain as an assault on the person's life and sense of identity. In addition, Tang et al [9] have demonstrated that, in chronic pain patients, mental defeat emerged as the strongest predictor of pain interference, depression and psychosocial disability, whereas catastrophizing was the best predictor of sleep interference, anxiety and functional disability.

Despite mental defeat appearing to be a relevant construct in chronic pain [6], it has not been previously assessed in fibromyalgia (FM), one of the most prevalent chronic pain syndromes. Another reason to assess MD in FM is that PTSD and depression, the two psychiatric disorders in which MD was originally studied, are frequently comorbid in FM and even can influence its outcome [10,11].

The aim of this paper is to validate the Spanish version of Pain Self Perception Scale (PSPS-Spanish) in patients with FM. Another secondary objective is to confirm whether mental defeat is a concept that differs from pain catastrophizing in patients with FM.

## Methods

### Translation process

Permission to translate and validate the PSPS was obtained from the original authors [6]. Two Spanish-speaking content experts (one psychiatrist and one psychologist) and one Spanish-speaking linguistic expert independently translated the PSPS into Spanish. Then, the experts evaluated the Spanish versions, and any discrepancies were discussed and rectified by consensus. A professional Spanish-English translator with no knowledge of the original English-language questionnaire independently back-translated the combined Spanish language questionnaire into English. The two content experts then compared the original English version to the backtranslated questionnaire. We have followed the usual guidelines for cross-cultural adaptations [12]. This paper is part of broader research on psychological constructs in fibromyalgia and their validation in Spanish [13-15].

### Participants

Sample size was calculated according to the recommended 10:1 ratio of number of subjects to number of test items [16]. The sample comprised consecutive patients with FM recruited from primary care settings, who were assessed at the Somatoform Disorders/FM Unit at Miguel Servet University Hospital, Zaragoza, Spain, during 2008. In order to be included in the study, patients had to fulfill the American College of Rheumatology criteria for primary FM [17], according to a diagnosis made by a Spanish National Health Service rheumatologist. The exclusion criteria were medical or psychiatric disorders that impede the patient to answer correctly the questionnaire, predominance of CFS symptoms, and poor knowledge of Spanish language. In 16 cases, CFS symptoms predominated, resulting in the exclusion those patients from the study. The study questionnaires and protocol were approved by the Ethical Committee of the regional health authority, and the patients signed a consent form attesting to their willingness to participate.

### Assessments

#### Pain Self Perception Scale, Spanish version (PSPS-Spanish)

This is the questionnaire to be validated. It has 24 items, with 11 items adapted from the PTSD mental defeat scale [3,4] and 13 items adapted from the depression defeat scale [5]. Respondents are asked to indicate to what extent each of the 24 statements applied to their experience "during a recent episode of intense pain". These statements are to be rated on a 5-point scale (0 = "Not at all/Never," 1 = "Very little," 2 = "Moderately," 3 = "Strongly," 4 = "Very strongly"), generating a total score ranging from 0 to 96. The original English-language version of this questionnaire has been demonstrated to have adequate psychometric properties [6].

#### Fibromyalgia Impact Questionnaire (FIQ)

The FIQ is a 10-item self-report questionnaire that measures the health status of patients with FM [18]. The first item focuses on patients' ability to perform physical activities. The following two items require the patients to indicate the number of days in the past week they felt good and how many days of work they missed. The remaining seven items concern the ability to work, pain, fatigue, morning tiredness, stiffness, anxiety, and depression and are measured with the visual analogue scale (VAS). In the present study we used a Spanish version of the FIQ that has been translated and validated [19].

#### Hospital Anxiety and Depression Scale (HADS)

The HADS [20] is a self-report scale that screens for the presence of depression and anxiety in patients with "medical conditions". It comprises 14 items that are rated on a 4-point Likert-type scale, and it is appropriate for use in community and hospital settings. Two subscales, HADS-Dep and HADS-Anx, independently assess depression and anxiety. The HADS was previously validated in a Spanish population [21]. HADS was selected for use in the present study because it is considered one of the best questionnaires for assessing depression and anxiety in patients with pain disorders [22].

#### Pain Visual Analogue Scale (PVAS)

The PVAS was designed to allow a subjective assessment of pain. A Visual Analogue Scale is usually a 10-cm horizontal line, with perpendicular lines on the edges, defined as the extreme limits of pain experience. The anchor points at each end are labeled as "No pain" (accompanied by the number "0") at one end and "maximum pain ever experienced" (accompanied by the number "100") at the other end. Previous studies have demonstrated PVAS to have adequate psychometric properties [23].

#### Pain Catastrophizing Scale (PCS)

The PCS is a 13-item self-report questionnaire that comprises three dimensions: (a) rumination, (b) magnification and (c) helplessness. Its validity and reliability have been previously reported [24]. Our group was responsible for validating the Spanish version of this questionnaire (15). There is no established "cut-off" point because pain catastrophizing is considered a personality trait distributed in a continuous way in the general population.

#### Standardized Polyvalent Psychiatric Interview (SPPI) [25]

This is a psychiatric interview developed by our group for the multiaxial assessment of psychiatric morbidity in medical patients. It permits the use of different diagnostic criteria, including DSM-IV and International Classification of Diseases, Tenth Revision (ICD-10). It was used to diagnose psychiatric comorbidity in the sample.

### Statistical analysis

In order to determine the suitability of the data for principal components analysis, the Kaiser-Meyer-Olkin Measure of Sampling Adequacy (KMO) [26] and Bartlett's Test of Sphericity [27] were calculated. Internal consistency was determined using Cronbach's alpha and item-total correlation coefficients. A principal components analysis was then performed to determine whether the 24 items in the questionnaire could be combined into separate components reflecting different aspects of mental defeat. Varimax rotation was performed to minimize the complexity of loadings for each component. Criterion validity of the PSPS-Spanish was examined by calculating the correlations between the total PSPS-Spanish score with the other questionnaires, using Pearson's *r *correlation coefficient. To confirm that pain catastrophizing and mental defeat are different constructs, a partial correlation analysis between PSPS-Spanish and the other scales controlling pain catastrophizing was performed. Test-retest reliability, evaluated with the intraclass correlation coefficient, was assessed for the 1 to 2-week follow-up interval, during which time the patients did not change baseline treatment. All statistical analyses were performed with SPSS software, Release 15 (SPSS Inc. Chicago, Illinois) except for the principal component analysis, which was performed with LISREL software, version 8.30 (Scientific Software International, Inc. Lincolnwood, Illinois).

## Results

### Translational issues

Differences between the original English-language version and the back-translated version of the PSPS-Spanish can be seen as Additional file 1. Most of the twenty-four items of the PSPS-Spanish were back-translated identically to or with minor differences with the original questionnaire. The final Spanish version is shown in Table 1.

**Table 1:** Final Spanish version of the ESCALA DE AUTOPERCEPCIÓN DEL DOLOR

1.- Me sentí derrotado por la vida	0 - 1 - 2 - 3 - 4
2.- Sentí que había perdido mi lugar en el mundo	0 - 1 - 2 - 3 - 4

3.- Sentí que la vida me había tratado como a un saco de boxeo	0 - 1 - 2 - 3 - 4

4.- Me sentí impotente	0 - 1 - 2 - 3 - 4

5.- Sentí que me habían arrancado la confianza a golpes	0 - 1 - 2 - 3 - 4

6.- Me sentí incapaz de manejarme con las situaciones que la vida me enviaba	0 - 1 - 2 - 3 - 4

7.- Sentí que me había hundido hasta el fondo de la escalera	0 - 1 - 2 - 3 - 4

8.- Sentí que me habían dejado completamente inútil	0 - 1 - 2 - 3 - 4

9.- Sentí que era uno de los perdedores de la vida	0 - 1 - 2 - 3 - 4

10.- Sentí que me había rendido	0 - 1 - 2 - 3 - 4

11.- Me sentí fuera de combate	0 - 1 - 2 - 3 - 4

12.- Sentí que había perdido batallas importantes en la vida	0 - 1 - 2 - 3 - 4

13.- Sentí que no me quedaban fuerzas para luchar	0 - 1 - 2 - 3 - 4

14.- Sentí que estaba perdiendo mi fuerza de voluntad	0 - 1 - 2 - 3 - 4

15.- Ya no me importaba lo que me pudiese ocurrir	0 - 1 - 2 - 3 - 4

16.- Me sentí derrotado	0 - 1 - 2 - 3 - 4

17.- Sentí que era menos que un ser humano	0 - 1 - 2 - 3 - 4

18.- Tal como yo lo veía, me había rendido	0 - 1 - 2 - 3 - 4

19.- Me sentí destrozado como persona	0 - 1 - 2 - 3 - 4

20.- Sentí que me quería morir	0 - 1 - 2 - 3 - 4

21.- Sentí que había perdido mi resistencia emocional	0 - 1 - 2 - 3 - 4

22.- Me sentí como un objeto	0 - 1 - 2 - 3 - 4

23.- Me sentí completamente a merced de lo que me ocurría	0 - 1 - 2 - 3 - 4

24.- Me sentí humillado y que había perdido mi sentido de la dignidad	0 - 1 - 2 - 3 - 4

Este cuestionario trata sobre cómo se siente durante un episodio reciente de dolor. En él encontrará un número de afirmaciones que describen los pensamientos y sentimientos que la gente a veces experimenta cuando sufre un episodio de dolor intenso. Por favor, puntúe la intensidad con las que estas afirmaciones se aplican a su experiencia durante el episodio de dolor intenso marcando con un círculo el número apropiado. No hay respuestas correctas o equivocadas. Por favor, recuerde que este cuestionario se refiere a cómo se siente usted durante los episodios de dolor intenso.

### Characteristics of the sample

Sixteen patients were ruled out from the study because CFS symptoms predominate. Of 253 potential subjects, three (1.1%) declined to participate. None of the participants were ruled out because of the exclusion criteria. The final study sample consisted of 250 patients, 229 (91.6%) women and 21 (8.4%) men, aged 24-61 (mean 44.9, SD: 7.2 years), all self-described as White European. Ratio women: men are quite more frequent in the sample reflecting a similar ratio in the prevalence of FM in either gender. On average, the patients who participated in the study had suffered from FM for 7.9 years (range 1-20; SD: 2.3 years), and 122 (48.8%) had been granted an invalidity pension. Most of the patients (231; 92.4%) were taking one or more prescription drugs. More than half of the patients (N = 131; 52.4%) suffered from some form of psychiatric morbidity assessed with SPPI, mainly depression and anxiety. A group of 21 patients (8.4%) were also diagnosed with PTSD.

### Distribution of total scores

The distribution of PSPS-Spanish total score (M = 33.4, SD = 26.7, Minimum = 0, Maximum = 94) did not differ significantly from a normal symmetric distribution (skewness = 0.66, SE = 0.19; kurtosis = - 0.74; SE = 0.35), although there was a slight positive skew. There were no significant differences in scoring between men (M = 32.4, SD = 27.8) and women (M = 34.2, SD = 25.9). However, the subsample of patients with FM and PTSD showed significantly higher scores in PSPS (M = 48.3, SD = 18.5) compared with the subgroup of patients with FM but without PTSD (M = 31.2, SD = 28.3) (*t *= 2.98, *df *= 248, *p *< 0.01). Mean and SD scores of the Spanish versions of the instruments used are summarized in Table 2. There was not significant association between PSPS total score and most demographic characteristics including gender, age, marital status, duration of pain, education level or work status, as can be seen in Table 3.

**Table 2:** Mean and SD scores of the Spanish versions of the instruments used

Instruments (range)	Mean	SD
PSPS (0-96)	33.4	26.7

FIQ (0-100)	70.8	15.2

HADS-anx (0-21)	11.8	4.2

HADS-dep (0-21)	12.5	4.7

PVAS (0-100)	73.1	14.9

PCS (0-52)	30.8	11.7

Abbreviatures: PSPS: Pain Self-Perception Scale; FIQ: Fibromyalgia Impact Questionnaire; HADS-anx: Hospital Anxiety Depression Scale, anxiety subscale; HADS-dep: Hospital Anxiety Depression Scale, depression subscale; PVAS: Pain Visual Analogue Scale; PCS: Pain Catastrophizing Scale

**Table 3:** Association between the Spanish version of PSPS and demographic parameters

Demographic parameters	Association	Significance
Gender	0.113	*p *= 0.192

Age	0.076	*p *= 0.653

Marital status	0.214	*p *= 0.342

Education level	0.198	*p *= 0.411

Duration of pain	-0.096	*p *= 0.604

Work status	0.123	*p *= 0.093

### Face validity

For assessing face validity a subsample (N = 150) of the validation study sample were randomly selected. They were asked whether they thought that the test could adequately measure the effects of pain as an assault on the person's life and sense of identity. A total of 95.3% patients (143 out of 150) agreed.

### Internal consistency

Cronbach's α calculation for the 24 items in the PSPS-Spanish was 0.90 (95% CI: 0.87-0.93), indicating a high degree of internal consistency. Item-total *r *correlation coefficients ranged between 0.68 and 0.86; median = 0.80) (Table 4).

**Table 4:** One-factor solution from Principal Component Analysis of the PSPS-Spanish

PSPS Item			Factor loading		
**"Because of the pain...**	**Factor 1**	**Factor 2**	**M**	**SD**	**Item-total *r***

19.- I felt destroyed as a person	**0.86**	0.04	1.21	1.43	0.86

16.- I felt defeated	**0.86**	-0.05	1.39	1.47	0.86

5.- I felt that my confidence had been knocked out of me	**0.85**	0.02	1.81	1.39	0.85

24.- I felt humiliated and that I was losing my sense of inner dignity	**0.85**	-0.08	1.32	1.41	0.85

3.- I felt that life had treated me like a punching bag	**0.83**	0.14	1.17	1.52	0.83

1.- I felt defeated by life	**0.83**	0.09	1.42	1.57	0.83

14.- I felt I was losing my will power	**0.82**	-0.12	1.28	1.45	0.81

9.- I felt I was one of life's losers	**0.82**	0.17	1.09	1.33	0.82

23.- I felt completely at the mercy of what was happening to me	**0.81**	0.13	1.21	1.37	0.81

12.- I felt I had lost important battles in life	**0.81**	-0.07	1.34	1.43	0.81

2.- I felt that I had lost my standing in the world	**0.80**	-0.21	1.24	1.32	0.80

15.- I didn't care what happened to me anymore	**0.80**	0.19	1.18	1.38	0.80

20.- I felt like I wanted to die	**0.80**	0.20	0.96	1.24	0.80

13.- I felt that there was no fight left on me	**0.79**	0.26	1.21	1.39	0.79

6.- I didn't feel able to deal with things that life threw at me	**0.79**	-0.25	1.08	1.33	0.78

18.- In my mind, I gave up	**0.77**	0.28	1.10	1.24	0.77

21.- I felt like I was losing my inner resistance	**0.77**	-0.30	1.29	1.38	0.77

10.- I felt that I had given up	**0.76**	0.26	0.96	1.26	0.76

8.- I felt completely knocked out of action	**0.76**	0.32	1.89	1.52	0.76

11.- I felt down and out	**0.74**	0.35	1.27	1.42	0.74

17.- I felt less like a human being	**0.72**	-0.27	0.91	1.21	0.72

4.- I felt powerless	**0.72**	0.37	1.67	1.45	0.71

7.- I felt I had sunk to the bottom of the ladder	**0.70**	0.39	1.34	1.39	0.70

22.- I felt like an object	**0.69**	**-0.43**	0.92	1.14	0.68

### Test-retest reliability

The response to the PSPS-Spanish provided by a random subsample of 75 patients with fibromyalgia (gender female: 70, 93.3%; age: mean 43.8 years, SD: 7.4 years; duration of the disorders: mean 7.6 years SD: 2.5 years; 39 (52%) granted an invalidity pension) showed satisfactory temporal stability of the scale over a 1-2 week interval, during which the patients did not change baseline treatment. The test-retest correlation assessed with the intraclass correlation coefficient was 0.78.

### Principal components analysis

The KMO was found to be 0.81, which exceeds the recommended minimum value of 0.60 [26]. Bartlett's Test of Sphericity was highly significant (χ2 = 753, *p *< 0.001), supporting the suitability of the data for principal components analysis [26]. A one-component solution was extracted using the Kaiser-Guttman rule (eigenvalues > 1.0) [28]. Results of the principal components analysis are shown in Table 3. It yielded two factors with eigenvalues greater than 1. The first factor, with an eigenvalue of 13.8, explained 61.4% of the variance; the second factor, with an eigenvalue of 1.9, explained only 7.3% of the total variance. All 24 items of the PSPS had high loadings on the first factor, ranging from 0.69 to 0.86. When factor loading smaller than 0.4 was suppressed, there was only 1 item loading on both factors (item 22). Therefore, item 22 is considered a "crossloading" item because it loaded high on more than one factor. With these results and a visual inspection of the scree plot, a one-factor solution is considered the most appropriate.

### Intercorrelations between PSPS-Spanish, depression, anxiety, pain, global function and pain catastrophizing

The total PSPS-Spanish score was significantly associated with all the questionnaires assessed (Table 5): HADS-dep (*r *= 0.63, *p *< 0.001), HADS-anx (*r *= 0.57, *p *< 0.001), pain assessed with PVAS (*r *= 0.42, *p *< 0.001), global function assessed with FIQ (*r *= 0.41, *p *< 0.001), and pain catastrophizing (*r *= 0.40, *p *<0.001). Total SPSS in the subsample of patients with fibromyalgia and PTSD also show a significant association (*p *< 0.001) with all the questionnaires having similar Pearson's *r *values to those of the whole sample: HADS-dep (*r *= 0.61), HADS-anx (*r *= 0.55), PVAS (*r *= 0.44) and pain catastrophizing (*r *= 0.42). However, PSPS correlation with FIQ is much more important in this subsample (*r *= 0.63).

**Table 5:** Interrelationship between mental defeat (PSPS), Pain, Anxiety, Depression, Catastrophizing and FIQ in patients with fibromyalgia

	1	2	3	4	5	6
1.- Mental defeat (PSPS)	----------------					

2.- Global function (FIQ)	0.41***	----------------				

3.- Depression (HADS-Dep)	0.63***	0.54***	--------------			

4.- Anxiety (HADS-Anx)	0.57***	0.52***	0.55***	----------------		

5.- Pain (PVAS)	0.42***	0.49***	0.42***	0.21*	----------------	

6.- Pain Catastrophizing (PCS)	0.40***	0.62***	0.53***	0.28*	0.44***	-------------------

*p < 0.05;

**p < 0.01;

***p < 0.00

To confirm that pain catastrophizing and mental defeat are different constructs, a partial correlation analysis was performed between PSPS-Spanish and the other scales controlling pain catastrophizing. The PSPS-Spanish score was significantly associated with all the questionnaires assessed, even those controlling pain catastrophizing: HADS-dep (*r *= 0.51, *p *< 0.001), HADS-anx (*r *= 0.48, *p *< 0.001), pain assessed with PVAS (*r *= 0.39, *p *< 0.001) and global function assessed with FIQ (*r *= 0.38, *p *< 0.001).

## Discussion

This study was undertaken to develop a Spanish language version of the PSPS and to assess its psychometric properties in FM patients. Additionally, one of its aims was also to confirm whether or not mental defeat and pain catastrophizing are overlapping concepts in patients with FM. The psychological construct that assesses PSPS, mental defeat, is expected to be important in predicting the outcome and in order to plan more effective psychological treatments in patients with chronic pain, including those with FM.

The sample of patients with FM in this study presents the expected demographic and clinical characteristics of this disorder: middle-aged women with several years of duration of the disorder, and with frequent psychiatric comorbidity and on invalidity pensions [29]. The prevalence of PTSD found was similar to that described in previous papers [30]. The translation process was relatively straightforward with only small differences between the original and the back-translated version of the questionnaire.

The psychometric properties of the PSPS-Spanish in patients with FM were similar to those of the original English-language validation study in chronic pain patients [6]. Internal consistency reported in our study was not as high as that reported by Tang et al. [6] (Cronbach's α = 0.90 in our study compared to 0.98). A Cronbach's α score of 0.90 may be more desirable, as it is well above the acceptable limit of 0.70 [31]. A very high Cronbach's α score indicates some degree of redundancy. This has been acknowledged by Tang et al [6], who suggest the need to develop a shorter version of the questionnaire. This may not be necessary in patients with FM. Test-retest reliability was also satisfactory.

Similarly to the initial validation study [6], the principal components analysis revealed a one-component solution emphasizing that this questionnaire is a monolithic measure. These data could be expected because FM is a subgroup of chronic pain patients and the selected sample included patients for who pain, and not fatigue, was the predominant symptom. Construct validity was evidenced by significant correlations between the PSPS-Spanish with all the questionnaires used in the validation process. As expected, and was observed in the original study, PSPS correlates with anxiety, depression and pain, but the strength of the relationship was within the moderate range (*r *= 0.42-0.63). We have included one measure of global function, the FIQ, and PSPS-Spanish correlates with this in a higher range in the subsample of patients with FM that also suffer from PTSD (*r *= -0.63) than in the whole sample (*r *= -0.41). This is coherent with other studies that demonstrate MD as a predictor of severity and chronicity in PTSD patients [1,2]. MD is expected to be much more important in the outcome and therapeutical approach taken with this subsample. Finally, another remarkable finding is the moderate correlation between PTSD and PCS (*r *= 0.40), suggesting that MD and pain catastrophizing are psychological constructs that are related but distinguishable from each other. This finding is also emphasized because MD measured by PSPS-Spanish is associated with depression, anxiety, pain and global function even controlling pain catastrophizing. One limitation of this study is that we did not assess the questionnaire in several populations, including healthy individuals. As this was undertaken in the original validation study we did not consider it necessary.

## Conclusion

The present study indicates that the Spanish version of the measure of MD, the Pain Self-Perception Scale, has good psychometric properties, including high levels of internal consistency and test-retest reliability. The one-factor structure is the same as that was found in the original validation study. MD seems to be more important for global function in patients with FM and with comorbid PTSD. MD and pain catastrophizing are different psychological constructs in patients with FM and also in chronic pain patients in general, as have been previously demonstrated [9].

## Competing interests

The authors declare that they have no competing interests.

## Authors' contributions

JGC, BR, MG and JVL conceived the study design. YLdH and MA collected the data, JGC and BR conducted the statistical analysis, and all authors interpreted the results, drafted the manuscript, and read and approved the final manuscript.

## Pre-publication history

The pre-publication history for this paper can be accessed here:

http://www.biomedcentral.com/1471-2474/11/255/prepub

## Supplementary Material

Additional file 1 **Back-translated version of the PSPS-Spanish**. This file compares the original English version of the PSPS questionnaire and the back translated version of the PSPS. Click here for file
