# Supplementary material for: Validation of a Spanish language version of the pain self-perception scale in patients with fibromyalgia
Source: BMC Musculoskelet Disord. 2010 Nov 4;11:255. doi: 10.1186/1471-2474-11-255 (PMC2991283; doi:10.1186/1471-2474-11-255)
Supplement: Additional file 1 — Back-translated version of the PSPS-Spanish. This file compares the original English version of the PSPS questionnaire and the back translated version of the PSPS. [file 1471-2474-11-255-S1.DOC]

Annex 1. Original PSPC scale compared to the back-translated PSPC version.

PAIN SELF-PERCEPTION SCALE (original) PAIN SELF-PERCEPTION SCALE (back-translated)

1.- I feel defeated by life 1.- I feel defeated by life

2.- I felt that I had lost my standing in the world 2.- I felt that I had lost my place in the world

3.- I felt that life had treated me like a punchbag 3.- I felt that life had treated me like a punching bag

4.- I felt powerless 4.- I felt powerless

5.- I felt that my confidence had been knocked out of me 5.- I felt that my confidence had been beaten out of me

6.- I didn’t feel able to deal with things that life threw at me 6.- I feel unable to handle the situations life sent me

7.- I feel that I had sunk to the bottom of the ladder 7.- I feel that I had sunk to the bottom of the ladder

8.- I felt completely knocked out of action 8.- I felt completely put out of action

9.- I felt that I was one of life’s losers 9.- I felt that I was one of life’s losers

10.- I felt that I had given up 10.- I felt that I had given up

11.- I felt down and out 11.- I felt down and out

12.- I felt I had lost important battles in life 12.- I felt I had lost important battles in life

13.- I felt that there was no fight left in me 13.- I felt that there was no fight left in me

14.- I felt I was losing my will power 14.- I felt I was losing my will power

15.- I didn’t care what happened to me anymore 15.- I did not care about what could happen to me

16.- I felt defeated 16.- I felt defeated

17.- I felt less like a human being 17.- I felt less like a human being

18.- In my mind, I gave up 18.- From my point of view, I gave up

19.- I felt destroyed as a person 19.- I felt destroyed as a person

20.- I felt like I wanted to die 20.- I felt like I wanted to die

21.- I felt like I was losing my inner resistance 21.- I felt like I was losing my emotional resistance

22.- I felt like an object 22.- I felt like an object

23.- I felt completely at the mercy of what was happening to me 23.- I felt completely at the mercy of what was happening to me

24.- I felt humiliated and that I was losing my sense of inner dignity 24.- I felt humiliated and that I was losing my sense of inner dignity
